# Mepolizumab reduces exacerbations in patients with severe eosinophilic asthma, irrespective of body weight/body mass index: meta-analysis of MENSA and MUSCA

**DOI:** 10.1186/s12931-019-1134-7

**Published:** 2019-07-30

**Authors:** Frank C. Albers, Alberto Papi, Camille Taillé, Daniel J. Bratton, Eric S. Bradford, Steven W. Yancey, Namhee Kwon

**Affiliations:** 1 Respiratory Medical Franchise, GSK, Research Triangle Park, NC, USA; 2 Research Center on Asthma and COPD, University of Ferrara, Ferrara, Italy; 3 Assistance Publique-Hôpitaux de Paris, Hôpital Bichat, Service de Pneumologie et Centre de Référence des Maladies Pulmonaires Rares, INSERM UMR1152, Paris, France; 4 Clinical Statistics, GSK, Stockley Park, Uxbridge, UK; 5 Respiratory Therapeutic Area, GSK, Research Triangle Park, NC USA; 6 Respiratory Medical Franchise, GSK, Brentford, Middlesex, UK; 7 Present address: Avillion US Inc., Northbrook, IL USA

**Keywords:** Asthma, Asthma pharmacology, Body mass index, Body weight, Mepolizumab

## Abstract

**Background:**

We assessed the efficacy of the licensed mepolizumab dose (100 mg subcutaneously [SC]) in patients with severe eosinophilic asthma according to body weight/body mass index (BMI).

**Methods:**

This was a post hoc individual patient-level meta-analysis of data from the Phase 3 studies MENSA (MEA115588/NCT01691521) and MUSCA (200862/NCT02281318). Patients aged ≥12 years with severe eosinophilic asthma and a history of exacerbations were randomised to 4-weekly placebo, mepolizumab 75 mg intravenously (IV) or 100 mg SC (MENSA) or placebo or mepolizumab 100 mg SC (MUSCA) for 32 (MENSA) or 24 (MUSCA) weeks. The primary endpoint was the annual rate of clinically significant exacerbations; other outcomes included the proportion of patients with no exacerbations, lung function, St George’s Respiratory Questionnaire (SGRQ) and Asthma Control Questionnaire-5 (ACQ-5) scores and blood eosinophil counts. Analyses were performed by baseline body weight and BMI (≤60, > 60–75, > 75–90, > 90, < 100, ≥100 kg; ≤25, > 25–30, > 30, < 36, ≥36 kg/m^2^).

**Results:**

Overall, 936 patients received placebo or mepolizumab 100 mg SC. Across all body weight/BMI categories, mepolizumab reduced the rate of clinically significant exacerbations by 49–70% versus placebo. Improvements with mepolizumab versus placebo were also seen in lung function in all body weight/BMI categories except > 90 kg; improvements in SGRQ and ACQ-5 scores were seen across all categories.

**Conclusions:**

Mepolizumab 100 mg SC has consistent clinical benefits in patients with severe eosinophilic asthma across a range of body weights and BMIs. Data show that the fixed-dose regimen of mepolizumab is suitable, without the need for weight-based dosing.

**Trial registration:**

This manuscript is a post hoc meta-analysis of data from the Phase 3 studies MENSA and MUSCA. ClinicalTrials.gov, NCT01691521 (MEA115588; MENSA). Registered September 24, 2012. ClinicalTrials.gov, NCT02281318 (200862; MUSCA). Registered November 3, 2014.

**Electronic supplementary material:**

The online version of this article (10.1186/s12931-019-1134-7) contains supplementary material, which is available to authorized users.

## Background

Asthma affects an estimated 339 million people worldwide [1], with 5–10% of this population having severe asthma, characterised by frequent, persistent respiratory symptoms, despite the regular use of maintenance therapies and additional controllers [2]. Severe asthma is a heterogeneous condition, with a number of clinical phenotypic expressions including severe eosinophilic asthma [2, 3]. Patients with severe eosinophilic asthma often develop disease in adult life, experience recurrent exacerbations and characteristically show eosinophilic inflammation despite appropriate treatment [2, 3], highlighting a need for more targeted therapy.

Mepolizumab is a monoclonal antibody (mAb) that selectively targets interleukin (IL)-5 and inhibits eosinophilic inflammation [4, 5]. In Phase 3 clinical studies, mepolizumab treatment was associated with reduced rates of exacerbations, decreased oral glucocorticoid dependence and improved lung function, asthma control and health-related quality of life (HRQoL), compared with placebo [6–8]. Mepolizumab is approved in the US and Europe for use as an add-on treatment for patients with severe eosinophilic asthma, and the licensed dose for adults and adolescents, administered subcutaneously (SC), is fixed at 100 mg, regardless of body weight [4, 5]. A fixed-dose regimen is preferred since it reduces the likelihood of dosing errors and drug wastage compared with a treatment regimen in which the dose is determined according to body weight [9]. However, overweight and obese patients may display reduced responses to some treatments as a result of altered drug bioavailability [10, 11]. As such, it is important to explore the treatment response of fixed-dosed regimens across a wide spectrum of body compositions.

The aim of this post hoc meta-analysis of data from two Phase 3 clinical trials, MENSA (MEA115588/NCT01691521) and MUSCA (200862/NCT02281318) [7, 8], was to assess the efficacy of the licensed dose of mepolizumab (100 mg SC) versus placebo in patients with severe eosinophilic asthma according to body weight and body mass index (BMI).

## Methods

### Study design

This was a post hoc individual patient-level meta-analysis of data from two Phase 3, placebo-controlled, randomised, double-blind, parallel-group, multicentre studies, MENSA and MUSCA [7, 8], which assessed the licensed dose of mepolizumab (100 mg SC) in patients with severe eosinophilic asthma. Results of these studies have been reported previously [7, 8]. In brief, patients enrolled in MENSA were randomised (1:1:1) to receive mepolizumab 75 mg intravenously (IV), mepolizumab 100 mg SC or placebo, plus standard of care (high-dose inhaled corticosteroids [ICS] and another controller), every 4 weeks for 32 weeks. Patients enrolled in MUSCA were randomised (1:1) to receive mepolizumab 100 mg SC or placebo, plus standard of care, every 4 weeks for 24 weeks. This post hoc analysis reports data from patients who received placebo or mepolizumab 100 mg SC only. MENSA and MUSCA were conducted in accordance with the ethical principles of the Declaration of Helsinki, International Conference on Harmonisation Good Clinical Practice Guidelines, and applicable country-specific regulatory requirements.

### Patients

MENSA and MUSCA enrolled patients aged ≥12 years with severe eosinophilic asthma (blood eosinophil count: ≥300 cells/μL in the previous year; or ≥ 150 cells/μL at screening) who had a history of ≥2 exacerbations (requiring systemic corticosteroids) in the 12 months prior to screening despite regular treatment with high-dose ICS during the same period, plus additional controller medication(s) with or without oral corticosteroids (OCS) for ≥3 months. Neither study included specific body weight or BMI inclusion criteria. Data from patients who received ≥1 dose of either placebo or mepolizumab were included in this meta-analysis; this was the modified intent-to-treat population.

### Endpoints and assessments

The primary endpoint was the annual rate of clinically significant exacerbations (defined as a worsening of asthma that required the use of systemic corticosteroids and/or hospitalisation/emergency department visit). Secondary endpoints included the proportion of patients with no clinically significant exacerbations over the course of the study, change from baseline in pre-bronchodilator forced expiratory volume in 1 s (FEV_1_) at study end, change from baseline in St George’s Respiratory Questionnaire (SGRQ) total score at study end, the proportion of responders achieving a ≥ 4-point reduction (minimal clinically important difference [MCID] [12]) from baseline in SGRQ total score at study end, change from baseline in Asthma Control Questionnaire (ACQ-5) score at study end, the proportion of responders achieving a ≥ 0.5-point reduction (MCID [13]) from baseline in ACQ-5 score at study end, and the change from baseline in blood eosinophil count at study end.

### Sample size and statistical analysis

Analyses were stratified by body weight (categories: ≤60, > 60–75, > 75–90 and > 90 kg [all endpoints]; thresholds: < 100 and ≥ 100 kg [primary endpoint only]) and BMI (categories: ≤25, > 25–30 and > 30 kg/m^2^ [all endpoints]; thresholds: < 36 and ≥ 36 kg/m^2^ [primary endpoint only]). Body weight categories were selected based on cut-offs used in analyses of previous mepolizumab studies. BMI categories were selected based on those generally used in clinical practice to define normal weight, overweight and obese.

The rate of exacerbations was analysed separately for each subgroup in each study using a negative binomial model, including the log of time on treatment as an offset variable. Continuous endpoints, including changes from baseline in pre-bronchodilator FEV_1_, SGRQ total score (scale 0–100, with higher scores indicating worse HRQoL), ACQ-5 score, and blood eosinophil count, were analysed using a mixed model repeated measures analysis. The proportion of patients with no clinically significant exacerbations and the proportions of SGRQ total score and ACQ-5 score responders were analysed using a logistic regression model. All model-based analyses were adjusted for treatment, number of exacerbations in the previous year (2, 3, ≥4), baseline maintenance OCS use, baseline pre-bronchodilator percent predicted FEV_1_ (except analysis of FEV_1_), geographical region, and baseline value of the analysis variable (where applicable). End-of-study treatment differences between mepolizumab 100 mg SC and placebo for each subgroup were combined across studies using an inverse variance weighted fixed-effects meta-analysis. The protocol for the meta-analysis is available on the GSK Clinical Studies Register (Study ID 208115) [14].

## Results

### Patient population

In total, 1136 patients who participated in the MENSA and MUSCA studies received ≥1 dose of study treatment and were included in the analysis. Of these, 936 were randomised to receive either placebo (*n* = 468) or mepolizumab 100 mg SC (*n* = 468). Patient demographics and baseline characteristics by body weight and BMI categories and thresholds are shown in Table 1. At baseline, patients had a mean weight of 78.1 kg and BMI of 28.0 kg/m^2^. In general, mean age was similar across body weight and BMI subgroups. Patients in the higher BMI and weight categories generally had worse SGRQ scores compared with those in the lower categories. Across subgroups, around one-quarter of patients were receiving OCS maintenance therapy at baseline.

**Table 1:** Summary of patient demographics and baseline characteristics by body weight and BMI

	Body weight						BMI					Total population (*N* = 936)
	Categories				Thresholds		Categories			Thresholds		
	≤60 kg (*N* = 144)	> 60–75 kg (*N* = 319)	> 75–90 kg (*N* = 288)	> 90 kg (*N* = 185)	< 100 kg (*N* = 819)	≥100 kg (*N* = 117)	≤25 kg/m^2^ (*N* = 323)	> 25–30 kg/m^2^ (*N* = 330)	> 30 kg/m^2^ (*N* = 283)	< 36 kg/m^2^ (*N* = 844)	≥36 kg/m^2^ (*N* = 92)	
Female, n (%)	125 (87)	198 (62)	149 (52)	76 (41)	499 (61)	49 (42)	188 (58)	179 (54)	181 (64)	482 (57)	66 (72)	548 (59)
Mean (SD) age, years	46.5 (18.7)	51.5 (14.0)	52.1 (12.0)	50.1 (11.3)	50.9 (14.2)	48.9 (11.3)	47.8 (16.4)	53.1 (13.0)	51.0 (11.0)	50.9 (14.1)	48.5 (11.7)	50.6 (13.9)
Mean (SD) duration of asthma, years	17.4 (12.1)	20.3 (14.9)	20.0 (14.5)	20.0 (14.9)	19.8 (14.4)	18.9 (14.6)	17.9 (13.8)	20.1 (14.4)	21.4 (14.9)	19.4 (14.2)	23.1 (15.4)	19.7 (14.4)
Clinically significant exacerbations in the previous year, n (%)												
Mean (SD)	3.4 (2.2)	3.2 (2.7)	3.0 (1.8)	3.2 (2.2)	3.2 (2.3)	3.2 (2.2)	3.2 (2.4)	3.1 (2.2)	3.2 (2.1)	3.2 (2.3)	3.3 (2.4)	3.2 (2.3)
2	72 (50)	187 (59)	171 (59)	91 (49)	464 (57)	57 (49)	181 (56)	188 (57)	152 (54)	472 (56)	49 (53)	521 (56)
3	28 (19)	61 (19)	52 (18)	49 (26)	160 (20)	30 (26)	60 (19)	73 (22)	57 (20)	170 (20)	20 (22)	190 (20)
≥ 4	44 (31)	71 (22)	65 (23)	45 (24)	195 (24)	30 (26)	82 (25)	69 (21)	74 (26)	202 (24)	23 (25)	225 (24)
Receiving maintenance OCS therapy at baseline, n (%)												
Yes	24 (17)	80 (25)	71 (25)	52 (28)	196 (24)	31 (26)	79 (24)	74 (22)	74 (26)	204 (24)	23 (25)	227 (24)
No	120 (83)	239 (75)	217 (75)	133 (72)	623 (76)	86 (74)	244 (76)	256 (78)	209 (74)	640 (76)	69 (75)	709 (76)
Mean (SD) % predicted pre-bronchodilator FEV_1_	62.8 (16.8)	59.4 (17.8)	59.1 (15.8)	57.7 (16.6)	59.5 (16.8)	59.4 (17.2)	61.4 (17.7)	58.7 (16.6)	58.3 (16.1)	59.7 (17.0)	58.1 (15.7)	59.5 (16.8)
Mean (SD) SGRQ total score	45.5 (20.6)	45.0 (18.6)	47.7 (19.1)	50.8 (17.5)	46.6 (19.2)	50.6 (17.1)	43.9 (19.5)	46.5 (18.5)	51.4 (18.1)	46.3 (19.0)	53.7 (17.4)	47.1 (18.9)
Mean (SD) ACQ-5 score	2.2 (1.3)	2.2 (1.1)	2.1 (1.2)	2.5 (1.2)	2.2 (1.2)	2.4 (1.1)	2.2 (1.1)	2.1 (1.2)	2.4 (1.2)	2.2 (1.2)	2.5 (1.2)	2.2 (1.2)
Geometric mean (SD of log) blood eosinophil count, cells/μL	370 (1.13)	320 (0.93)	300 (0.90)	360 (0.87)	320 (0.96)	360 (0.85)	350 (0.98)	310 (0.90)	310 (0.96)	320 (0.96)	330 (0.81)	330 (0.95)

Percentages may not add up to 100, due to rounding

*Abbreviations*: *ACQ* Asthma Control Questionnaire, *BMI* Body mass index, *FEV*_*1*_ Forced expiratory volume in 1 s, *OCS* Oral corticosteroid, *SD* Standard deviation, *SGRQ* St George’s Respiratory Questionnaire

### Primary endpoint

**Fig. 1:**
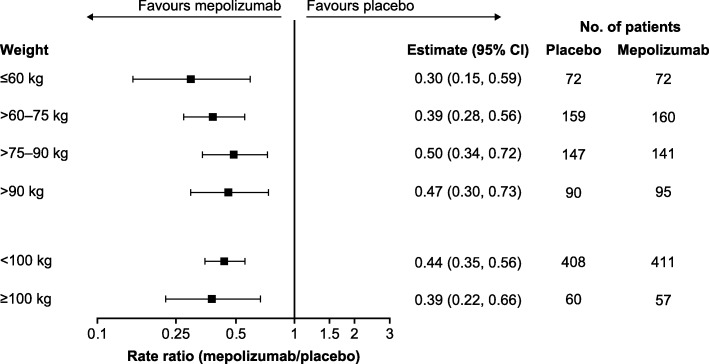
Ratio of the annual rate of clinically significant exacerbations. Mepolizumab 100 mg SC versus placebo. *CI* confidence interval, *SC* subcutaneous

**Fig. 2:**
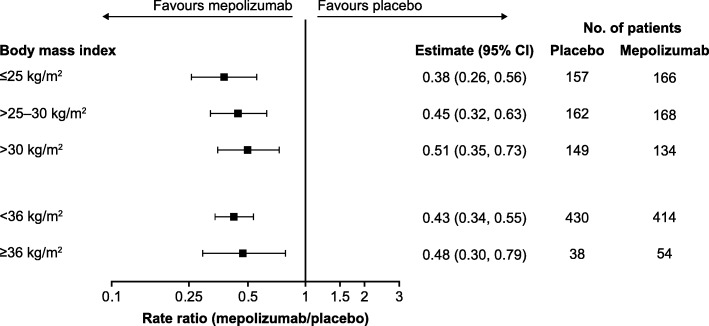
Ratio of the annual rate of clinically significant exacerbations. Mepolizumab 100 mg SC versus placebo. *BMI* body mass index, *CI* confidence interval, *SC* subcutaneous

Across all body weight categories and thresholds, mepolizumab 100 mg SC treatment was associated with reductions of 50–70% in the annual rate of clinically significant exacerbations compared with placebo (Fig. 1). Reductions of 49–62% in the annual rate of clinically significant exacerbations were also seen across BMI categories and thresholds with mepolizumab versus placebo (Fig. 2).

### Secondary endpoints

**Fig. 3:**
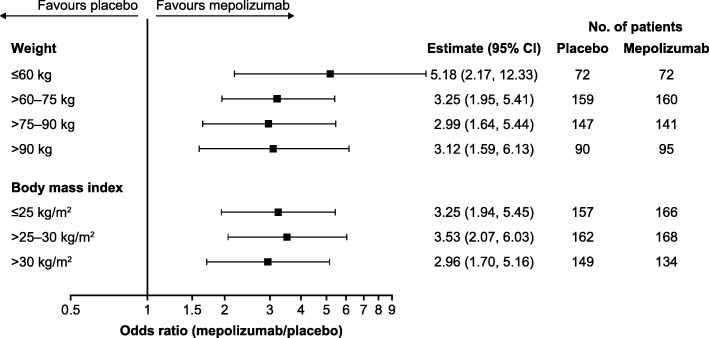
Proportion of patients experiencing no clinically significant exacerbation. Mepolizumab 100 mg SC versus placebo. *BMI* body mass index, *CI* confidence interval, *SC* subcutaneous

Regardless of body weight category, patients receiving mepolizumab 100 mg SC were more likely to experience no clinically significant exacerbations during the study period than those who received placebo, with odds ratios to placebo ranging between 2.99 (95% confidence interval [CI]: 1.64, 5.44) in the > 75–90 kg subgroup and 5.18 (95% CI: 2.17, 12.33) in the lowest weight subgroup of ≤60 kg (Fig. 3). Similar results were seen across BMI categories, with odds ratios to placebo ranging from 2.96 (95% CI: 1.70, 5.16) in patients in the highest BMI subgroup of BMI > 30 kg/m^2^ to 3.53 (95% CI: 2.07, 6.03) in those with a BMI > 25–30 kg/m^2^.

**Fig. 4:**
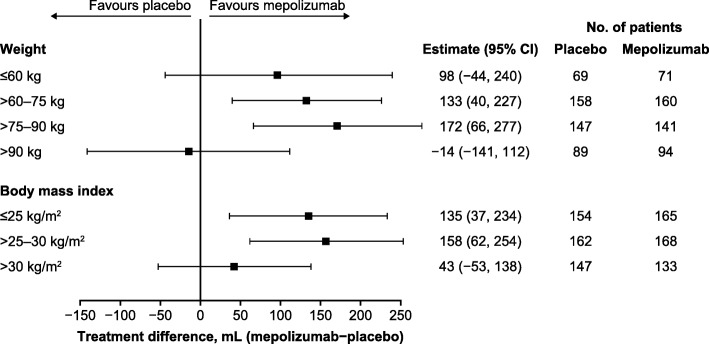
Change from baseline in pre-bronchodilator FEV_1_ (mL). Mepolizumab 100 mg SC versus placebo. *BMI* body mass index, *CI* confidence interval, *FEV*_*1*_ forced expiratory volume in 1 s, *SC* subcutaneous

Mepolizumab treatment resulted in an increase from baseline in pre-bronchodilator FEV_1_ versus placebo in patients with body weight ≤ 60, > 60–75 and > 75–90 kg (treatment difference ranged from 98 to 172 mL), but not in patients with body weight > 90 kg (treatment difference: − 14 mL) (Fig. 4). Across all BMI categories, mepolizumab treatment resulted in an increase from baseline in pre-bronchodilator FEV_1_ versus placebo, with a smaller effect in the highest BMI category (treatment difference ranged from 43 to 158 mL) (Fig. 4).

**Fig. 5:**
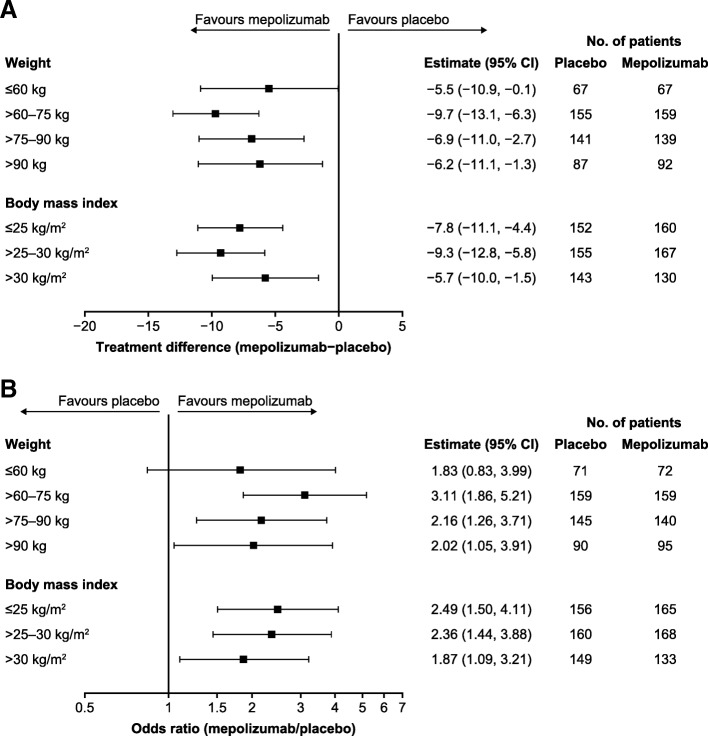
Change from baseline in SGRQ total score (**a**) and proportion of responders achieving a ≥ 4-point change from baseline in SGRQ total score (**b**). Mepolizumab 100 mg SC versus placebo. *BMI* body mass index, *CI* confidence interval, *SC* subcutaneous, *SGRQ* St George’s Respiratory Questionnaire

Improvements from baseline with mepolizumab versus placebo were observed in SGRQ total score at study end, irrespective of body weight or BMI category. Treatment differences ranged from − 5.5 to − 9.7 points across weight categories, and from − 5.7 to − 9.3 points across BMI categories (Fig. 5a). In addition, patients receiving mepolizumab treatment were more likely to achieve a clinically meaningful response of ≥4-point reduction (MCID) from baseline in SGRQ total score compared with patients receiving placebo, irrespective of body weight or BMI category (Fig. 5b).

**Fig. 6:**
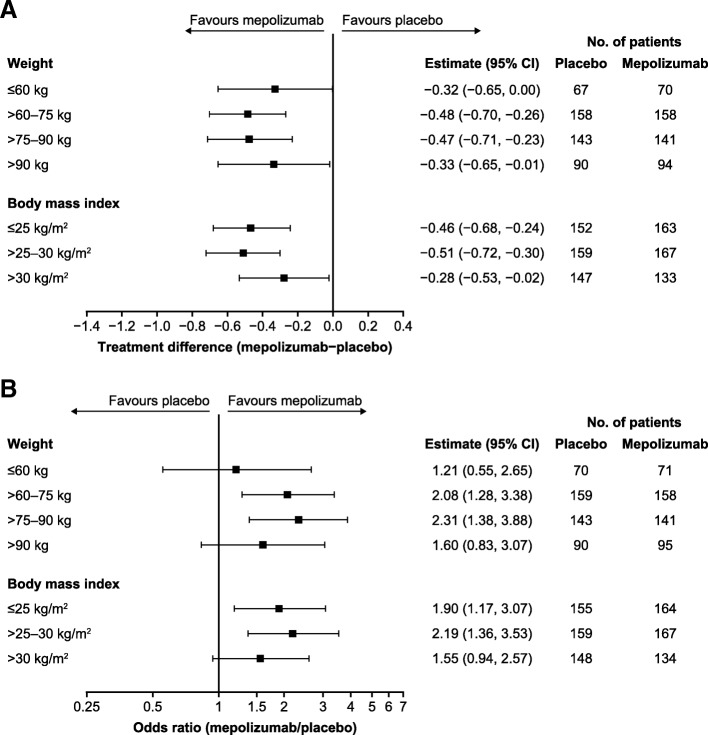
Change from baseline in ACQ-5 score (**a**) and proportion of responders achieving a ≥ 0.5-point change from baseline in ACQ-5 score (**b**). Mepolizumab 100 mg SC versus placebo. *ACQ-5* Asthma Control Questionnaire, *BMI* body mass index, *CI* confidence interval, *SC* subcutaneous

Mepolizumab was associated with improvements from baseline in ACQ-5 score at study end, compared with placebo, across all body weight categories (treatment difference ranged from − 0.32 to − 0.48 points) and all BMI categories (treatment difference ranged from − 0.28 to − 0.51) (Fig. 6a). Patients in all body weight categories were also more likely to achieve a clinically meaningful improvement of ≥0.5-point reduction from baseline in ACQ-5 score (MCID) when treated with mepolizumab versus placebo (odds ratio ranged from 1.21 to 2.31), as were patients across all BMI categories (odds ratio ranged from 1.55 to 2.19) (Fig. 6b).

**Fig. 7:**
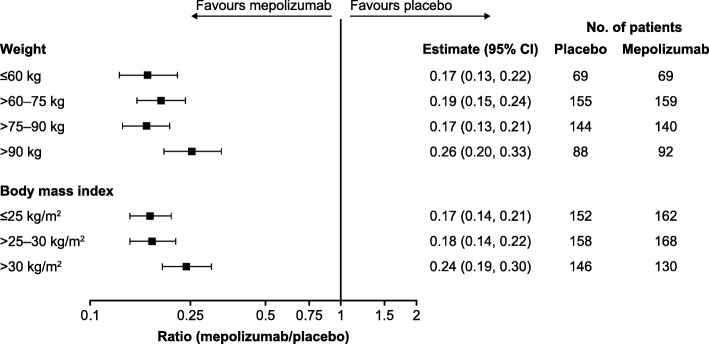
Ratio to baseline in blood eosinophil count. Mepolizumab 100 mg SC versus placebo. *BMI* body mass index, *CI* confidence interval, *SC* subcutaneous

Reductions from baseline in blood eosinophil count were seen in mepolizumab- versus placebo-treated patients across all body weight categories, with reductions ranging from 83% (ratio [95% CI]: 0.17 [0.13, 0.21]) in the > 75–90 kg subgroup to 74% (0.26 [0.20, 0.33]) in the > 90 kg subgroup (Fig. 7). Across BMI categories, mepolizumab treatment resulted in greater reductions from baseline in blood eosinophil count than placebo treatment, ranging from 83% (0.17 [0.14, 0.21]) in the ≤25 kg/m^2^ subgroup to 76% (0.24 [0.19, 0.30]) in the > 30 kg/m^2^ subgroup (Fig. 7).

## Discussion

In this post hoc meta-analysis of the MENSA and MUSCA studies, the licensed dose of mepolizumab (100 mg SC) showed consistent improvements versus placebo in exacerbation rate, HRQoL and asthma control, independent of patients’ body weight or BMI. These data demonstrate comparable efficacy of mepolizumab in patients with either high or low body weight/BMI, and confirm that dose-weight adjustments with mepolizumab are not required, thereby addressing queries regarding the need for higher mepolizumab doses in patients with high body weight/BMI. Our findings reinforce the suitability of a simple, fixed-dose regimen across all ranges of body weight/BMI in eligible patients.

Several novel biologic treatments approved for use in patients with differing severe asthma phenotypes have demonstrated reduced efficacy in patients who are obese. For example, a recent retrospective study in patients with severe asthma (*N* = 340) found that obesity may reduce the effectiveness of the anti-immunoglobulin E mAb omalizumab, independent of other asthma-influencing factors [15]. In addition, a post hoc pooled analysis of the Phase 3 SIROCCO and CALIMA trials demonstrated that the effects of the anti-IL-5 receptor mAb benralizumab on the annual rate of exacerbations and lung function in patients with severe eosinophilic asthma were less robust in obese individuals than in those who were of normal weight [16]. It is important to note that the DREAM study, which was a double-blind, placebo-controlled trial assessing mepolizumab in patients with severe eosinophilic asthma, showed no dosing effect over a 10-fold dose range of mepolizumab on exacerbation rate and other outcomes [17]. For this reason it was important to further investigate whether there was any relationship between mepolizumab efficacy and body weight/BMI in patients with severe eosinophilic asthma.

To date, the effect of mepolizumab 100 mg SC in patients with high body weight/BMI has not been extensively investigated. However, a post hoc analysis of the DREAM study suggested that mepolizumab remains efficacious in these individuals [18, 19]. In this analysis responder subgroups were identified using cluster analysis; the cluster demonstrating greatest response to mepolizumab was an obese, eosinophilic group with airway reversibility [19]. Moreover, a post hoc analysis of the MENSA and DREAM studies showed that treatment with mepolizumab (doses of 75, 250 or 750 mg IV or 100 mg SC) resulted in similar exacerbation rate reductions across weight categories of ≤60 kg, > 60–≤75 kg, > 75–≤90 kg and > 90 kg, with no discernible trend noted in exacerbation reductions with the 250 mg and 750 mg IV doses of mepolizumab, even at the higher weight categories (Additional file 1: Table S1) [20]. In the current analysis, mepolizumab treatment was associated with reduced exacerbation rates across all body weight and BMI categories, versus placebo. In addition, mepolizumab induced an increase from baseline in pre-bronchodilator FEV_1_ versus placebo in all body weight categories except > 90 kg. A similar trend was observed in the BMI categories. This is consistent with results from post hoc analyses of data from Phase 3 trials of benralizumab [16, 21]. For example, it has been shown that the increase in pre-bronchodilator FEV_1_ following benralizumab treatment was lower in patients with extremely high body weight (≥115 kg) compared with those with a body weight < 115 kg [21]. It is possible that mechanical factors causing airway restriction may be responsible for a reduced treatment response in obese patients [15], although it should also be noted that low patient numbers in the highest body weight category may have contributed to this observation in our analysis.

Consistent improvements in both SGRQ total score and ACQ-5 score were also seen with mepolizumab versus placebo across all body weight and BMI categories, including patients with body weight > 90 kg. Although the effects of mepolizumab on measures of HRQoL and asthma control have been reported several times previously in populations with severe eosinophilic asthma [7, 8], this is the first analysis to demonstrate a consistent effect on these parameters across all body weight and BMI categories. Given the particularly poor QoL and reduced asthma control in patients with asthma and comorbid obesity [22], this is a clinically important finding.

Also of clinical relevance is the consistent and sustained reduction in blood eosinophil count with mepolizumab versus placebo shown in this study, regardless of body weight/BMI. This finding builds upon a substantial body of evidence demonstrating mepolizumab-induced reductions in blood eosinophil levels and a correlation between reduced blood eosinophils and improvements in clinical parameters such as exacerbations and lung function [7, 8]. Of particular importance in this analysis, obesity was previously thought to be associated with a non-eosinophilic asthma phenotype that is typically unresponsive to steroids and therefore not considered to be eligible for treatment with mepolizumab [18]. However, more recently, elevated sputum IL-5 and submucosal and sputum eosinophils have been reported in obese patients with asthma [18, 23, 24]. The baseline patient characteristics in the current study are in line with this finding, with no obvious trend for lower blood eosinophil counts in patients with higher body weight or BMI, albeit in a population of patients who satisfied inclusion criteria pertaining to eosinophilic asthma. Together, these data suggest that patients with high body weight/BMI can have raised blood eosinophil levels and therefore be eligible for mepolizumab treatment, and further, that mepolizumab 100 mg SC can be efficacious in this population. One important consideration is the evidence that inflammatory biomarkers, including blood eosinophils, may be less predictive of airway eosinophilia in obese patients [25]. Nonetheless, the current analysis has demonstrated improvements in a range of clinical parameters in a population of patients with severe eosinophilic asthma across body weight and BMI categories.

Body weight typically influences the exposure of biologics [26]. However, whether this exposure difference translates into a meaningful efficacy difference also depends on the drug-to-target ratio. For omalizumab to adequately neutralise immunoglobulin E levels, a weight-based dosing strategy was deemed necessary [27]. By contrast, neutralisation of IL-5 levels with a fixed-dose regimen of mepolizumab 100 mg SC was found to be efficacious and sufficient over the expected body weight/BMI range in adults and adolescents. A previously reported analysis showed that the efficacy dose–response was consistent with that of the well-defined pharmacological dose–response, in which the half-maximal effect was estimated at 11 mg SC [28], which is well below the licensed dose of 100 mg SC. Unlike mepolizumab, the clinical development programme for reslizumab only investigated a mg/kg dosing regimen and reslizumab is therefore dosed according to body weight [29].

Combining data from two large randomised, controlled trials in the current analysis provided a large sample in which to determine the effect of mepolizumab across a range of body compositions. However, there are several limitations that should be considered. First, the post hoc nature of the analysis should be considered when interpreting the findings. In addition, the number of patients varied substantially between subgroups, with smaller patient numbers in the highest body weight and BMI subgroups. We also did not investigate whether there were any differences in safety findings between the subgroups, although previous studies have demonstrated that mepolizumab is well tolerated with minimal immunogenic potential [6–8]. Despite these limitations, our findings provide valuable insights into the use of mepolizumab in patients with severe eosinophilic asthma.

## Conclusions

In summary, results from this post hoc analysis of the MENSA and MUSCA studies demonstrate that mepolizumab 100 mg SC is associated with improvements in exacerbation rate, HRQoL and asthma control in patients with severe eosinophilic asthma, across a range of patient body weights and BMI categories. The reason for a lower improvement in FEV_1_ in the highest body weight category remains to be investigated. Nonetheless, our findings support the use of a simple, fixed-dose regimen of mepolizumab 100 mg SC for treating patients with severe eosinophilic asthma.

## Additional file

Additional file 1: **Table S1.** Rate ratio of clinically significant exacerbations by body weight in the MENSA and DREAM studies (intent-to-treat population). (PDF 590 kb)

## Data Availability

Anonymised individual participant data from the studies listed within this publication and their associated documents can be requested for further research from www.clinicalstudydatarequest.com.

## Abbreviations

ACQ-5: Asthma Control Questionnaire-5
BMI: Body mass index
FEV_1_: Forced expiratory volume in 1 s
HRQoL: Health-related quality of life
ICS: Inhaled corticosteroids
IL: Interleukin
IV: Intravenously
mAb: monoclonal antibody
OCS: Oral corticosteroids
SC: Subcutaneously
SGRQ: St George’s Respiratory Questionnaire
