# Supplementary material for: Mepolizumab reduces exacerbations in patients with severe eosinophilic asthma, irrespective of body weight/body mass index: meta-analysis of MENSA and MUSCA
Source: Respir Res. 2019 Jul 30;20:169. doi: 10.1186/s12931-019-1134-7 (PMC6664536; doi:10.1186/s12931-019-1134-7)
Supplement: Supplementary file 1 — Table S1. Rate ratio of clinically significant exacerbations by body weight in the MENSA and DREAM studies (intent-to-treat population). (PDF 590 kb) [file 12931_2019_1134_MOESM1_ESM.pdf]

Mepolizumab reduces exacerbations in patients with severe eosinophilic asthma, irrespective of body weight/body mass index: meta-analysis of MENSA and MUSCA (Albers *et al.*)

Contents

Supplementary Table S1 Rate ratio of clinically significant exacerbations by body weight in the MENSA and DREAM studies (intent-to-treat population).

Supplementary Table S1 Rate ratio of clinically significant exacerbations by body weight in the MENSA and DREAM studies (intent-to-treat population).

	Body weight categories			
	**≤60 kg**	**>60–75 kg**	**>75–90 kg**	**>90 kg**
**Exacerbation rate ratio, 95% CI, mepolizumab/placebo**				
**DREAM study**				
Mepolizumab 75 mg IV	n=25<br>0.67 (0.39, 1.14)	n=47<br>0.57 (0.33, 0.96)	n=52<br>0.58 (0.34, 0.98)	n=29<br>0.25 (0.13, 0.49)
Mepolizumab 250 mg IV	n=25<br>0.46 (0.25, 0.85)	n=44<br>1.09 (0.68, 1.74)	n=43<br>0.40 (0.23, 0.71)	n=40<br>0.44 (0.26, 0.75)
Mepolizumab 750 mg IV	n=18<br>0.21 (0.09, 0.48)	n=41<br>0.69 (0.41, 1.16)	n=48<br>0.45 (0.26, 0.79)	n=49<br>0.52 (0.32, 0.87)
**MENSA study**				
Mepolizumab 100 mg SC	n=41<br>0.32 (0.14, 0.75)	n=67<br>0.39 (0.23, 0.65)	n=55<br>0.58 (0.34, 1.00)	n=31<br>0.64 (0.33, 1.21)
Mepolizumab 75 mg IV	n=40<br>0.48 (0.20, 1.16)	n=50<br>0.36 (0.20, 0.66)	n=63<br>0.73 (0.44, 1.23)	n=38<br>0.62 (0.34, 1.13)

DREAM and MENSA were randomized, multicentre, double-blind, placebo-controlled trials.[1,2] Patients had a history of ≥2 exacerbations requiring systemic corticosteroid treatment in the previous year and evidence of eosinophilic inflammation. Patients enrolled in DREAM were randomised (1:1:1:1) to receive mepolizumab 75 mg IV (n=154), mepolizumab 250 mg IV (n=152), mepolizumab 750 mg IV (n=156) or placebo (n=159), plus standard of care, every 4 weeks for 52 weeks (totalling 13 infusions). Patients enrolled in MENSA were randomised (1:1:1) to receive mepolizumab 75 mg IV (n=191), mepolizumab 100 mg SC (n=194) or placebo (n=191), plus standard of care, every 4 weeks for 32 weeks.

CI, confidence interval; IV, intravenous; SC, subcutaneous.

References

1. Ortega HG, Liu MC, Pavord ID*, et al.* Mepolizumab treatment in patients with severe eosinophilic asthma. *N Engl J Med* 2014;371:1198-207.
2. Pavord ID, Korn S, Howarth P*, et al.* Mepolizumab for severe eosinophilic asthma (DREAM): a multicentre, double-blind, placebo-controlled trial. *The Lancet* 2012;380:651-59.
